# The effects of morning/afternoon surgeries on the early postoperative sleep quality of patients undergoing general anesthesia

**DOI:** 10.1186/s12871-022-01828-w

**Published:** 2022-09-10

**Authors:** Haitao Hou, Shujing Wu, Yuxue Qiu, Fenxiang Song, Liqin Deng

**Affiliations:** 1 Department of Anesthesiology, General Hospital of Ningxia Medical University, Yinchuan, 750004 Ningxia China; 2 Clinical College of Ningxia Medical University, Yinchuan, 750004 Ningxia China

**Keywords:** Morning and afternoon surgeries, Early postoperative sleep, General anesthesia, Circadian rhythm

## Abstract

**Objective:**

This study aimed to investigate the effects of morning and afternoon surgeries on the early postoperative sleep function in patients undergoing general anesthesia.

**Methods:**

Fifty nine patients, aged 18–60 years, American society of anaesthesiologists (ASA) grade I or II, Body mass index of 18.5–28 kg/m^2^, undergoing laparoscopic myomectomy under total intravenous anesthesia, were included in the study. These patients were divided into two groups according to the start time of anesthesia: morning surgery group (group A, 8:00–12:00) and afternoon surgery group (group P, 14:00–18:00). The sleep conditions of the two groups of patients were evaluated by the Athens Insomnia Scale (AIS) one day before and one day after the operation. A total score of > 6 was regarded as postoperative sleep disturbance. The incidences of sleep disturbance one day after the operation in two groups were compared. The bispectral Index assessed the patient’s total sleep duration, sleep efficiency, and overall quality of sleep from 21:00 to 6:00 on the first night after surgery. Plasma concentrations of melatonin and cortisol at 6:00 am 1 day before surgery, 1 day after surgery were measured by ELISA, and rapid random blood glucose was measured.

**Results:**

The total AIS score, overall quality of sleep, total sleep duration, and final awakening earlier than desired scores of the two groups of patients on the first night after surgery were significantly increased compared with preoperative scores (*P* < 0.01). In group P, the sleep induction and the physical and mental functioning during the day scores increased significantly after surgery compared with preoperative scores (*P* < 0.05). The postoperative AIS scores in group P increased significantly compared with those in group A (*P* < 0.01). The incidence of postoperative sleep disturbances (70.0%) in group P was significantly higher than that in group A (37.9%) (*P* < 0.05). Compared with group A, the total sleep duration under BIS monitoring in group P was significantly shorter, the sleep efficiency and the overall quality of sleep was significantly reduced (*P* < 0.01). Compared with those in group A, the level of melatonin on 1 d after surgery in group P was significantly decreased, and the level of cortisol in group P was significantly increased. There were no significant differences between the two groups in the levels of postoperative blood glucose and pain.

**Conclusion:**

Both morning and afternoon surgeries have significant impacts on the sleep function in patients undergoing general anesthesia, while afternoon surgery has a more serious impact on sleep function.

**Trial registration:**

ClinicalTrials, NCT04103528. Registered 24 September 2019—Retrospectively registered, http://www.ClinicalTrials.gov/ NCT04103528.

## Introduction

The circadian rhythm is an internal timing mechanism that is generated by the body to adapt to the external environment, which is closely related to the sleep–wake cycle of humans [1]. Patients are prone to changes in sleep pattern and overall quality of sleep after surgery, which is called postoperative sleep disturbances (POSD). It mainly includes a decrease in total sleep duration, disappearance of rapid eye movement sleep, a significant decrease in slow-wave sleep, a significant increase in light sleep, an increase in the number of awakenings, and highly fragmented sleep [2]. It aggravates postoperative pain and postoperative fatigue, increasing postoperative delirium and cardiovascular adverse events and even cause accidental death of patients [3]. It is not clear whether the early postoperative sleep function and the quality of postoperative recovery of patients undergoing general anesthesia are affected by morning and afternoon surgeries. This study aimed to observe the effects of morning and afternoon surgeries on the early postoperative sleep function in patients undergoing general anesthesia.

## Methods

This study is a prospective, randomized, control study, approved by the ethics committee of General hospital of Ningxia Medical University (approval number: 2018–247), and signed an informed consent form with the patient or his family.

### Subjects

Patients who underwent elective laparoscopic myomectomy under total intravenous anesthesia between October 2018 and January 2019 in our hospital, aged 18–60 years, American society of anaesthesiologists (ASA) grade I or II, Body mass index of 18.5–28 kg/m^2^, were included in the study.

Exclusion criteria were: patients who have combined ovarian diseases or underwent ovarian surgeries, diabetes mellitus, a preoperative Pittsburgh sleep quality index (PSQI) [4] score of ≥ 6 points, on long-term use of sedative drugs, hypnotics, and psychotropic drugs, on recent night shifts, flying across time zones, having an irregular lifestyle, patients who are unable to communicate due to mental illness or hearing, visual impairment, etc., and patients with sleep apnea syndrome. Elimination criteria were: patients voluntarily withdrawing from the study or were unable to complete the trial after the operation, those with more than 500 ml of intraoperative and postoperative bleeding, and the operation time was more than 2 h. Patients were divided into the morning surgery group (group A, 8:00–12:00) and the afternoon surgery group (P group, 14:00–18:00). Finally, 29 patients in Group A and 30 patients in Group Pwere included in the study by using random number table.

### Anesthesia methods

Patients in both groups were given total intravenous anesthesia. Patients were instructed to fast for 8 h before the operation and no pre-anesthesia medication was applied. After the patient entered the room, the upper extremity venous access was established and electrocardiogram, heart rate, noninvasive blood pressure, oxygen saturation_,_ and bispectral index (BIS) were monitored. Anesthesia induction: sufentanil 0.3 µg/kg, etomidate 0.2–0.4 mg/kg, rocuronium 0.6–0.8 mg/kg, and dexamethasone 10 mg were administrated intravenously, and 3 min later, a laryngeal mask airway was inserted into the area behind the mouth for mechanical ventilation. Respiratory parameters: VT 6–8 ml/kg, RR 12–16 times/min, I:E 1:2 and oxygen concentration is 40%. The respiratory rate was maintained at PetCO_2_ 35–45 mmHg during the operation. Anesthesia maintenance: remifentanil 0.1–0.3 µg·kg^−1^ min^−1^ and propofol 4–6 mg·kg^−1^ h^−1^ were continuously injected via an IV pump, rocuronium 10–25 mg was added to maintain muscle relaxation, and rocuronium was stopped 20 min before the end of the operation. Remifentanil and propofol were stopped at the end of the operation. Considering that dexmedetomidine affects sleep and may interfere with our final results, so this drug was not used in this study. The BIS was maintained at 40–60 during the operation. After the operation, the patient's spontaneous breathing was restored, and atropine 0.02 mg/kg and neostigmine 0.04 mg/kg were given to antagonize the residual effect of muscle relaxation. The laryngeal mask was removed after spontaneous breathing was V_T_ > 6 ml/kg and the consciousness and protective reflex were recovered. The patient was sent to the postanesthesia care unit (PACU). After being fully awake, the patient was sent back to the ward. Perioperative pain management: Parecoxib sodium 40 mg was injected intravenously 30 min before the operation. 0.75% ropivacaine was injected around the incision after the operation. Parecoxib sodium was applied every 12 h after surgery for postoperative analgesia. If the postoperative pain was > 3 points, intramuscular injection of Diclofenac Sodium 75 mg was carried out for remedial analgesia, if the patient had moderate to severe nausea or vomiting, tropisetron 2 mg was given intravenously (The NRS scale was used for postoperative pain and PONV (0–10 points, 0 points for no pain or PONV, 10 points for the most serious pain and PONV), > 3 indicates moderate or higher pain or PONV). Iatrogenic interference was minimized at night in the ward to ensure the patient’s rest [5].

### Observation indicators

The general data of the two groups of patients were recorded.

The Athens Insomnia Scale (AIS) was used to evaluate the sleep status of the two groups of patients one day before and one day after the operation. The AIS scale consists of eight items: the first five pertain to sleep induction, awakening during the night, final awakening earlier than desired, total sleep duration, and overall quality of sleep; while the last three refer to well-being, functioning capacity, and sleepiness during the day. Each item was recorded according to sleep conditions, rated 0–3 points (0 point means very good, 1 point means good, 2 points means poor, 3 points means very poor). The total AIS score (range, 0–24 points) was obtained by adding up the scores of the above items. The higher the score, the worse the sleep, a AIS score of > 6 points indicate sleep disturbances, < 4 points indicate no sleep disturbances, 4–6 points indicates suspicious sleep disturbances [6].

The BIS was used to monitor the total sleep duration, sleep efficiency, and overall quality of sleep of the two groups of patients on the first night (21:00–06:00) after surgery [7]. The BIS value was used to evaluate the total sleep duration (the time of BIS < 80), sleep efficiency (the ratio of the time of BIS < 80 to the monitoring time), and overall quality of sleep [the area under the BIS curve (BIS-AUC), the smaller the BIS-AUC, the better the overall quality of sleep].

The blood melatonin (MT), cortisol, and blood glucose levels were measured by ELISA in patients one day before and one day after the operation at 6 o'clock in the morning, blood collection at night will interfere with the patient's sleep, so collection is selected at 6:00 a.m.

### Statistical analysis

The postoperative AIS scores of the two groups of patients were taken as the main results. According to the pre-test data (*n* = 30, 15 cases in group A, 15 cases in group P), the incidence of POSD in group A (mean ± SD = 6.067 ± 2.071) was 47%, and the incidence of POSD in group P (mean ± SD = 7.867 ± 2.293) was 67%. A two-sided test was used (α = 0.05, β = 0.2), the required sample size in each group was at least 25 cases. Normally distributed measurement data were expressed as mean ± standard difference (mean** ± **SD); the paired *t*-test was used for comparisons within groups. Abnormally distributed measurement data and grade data were expressed in the median and interquartile range. Wilcoxon rank-sum test was used for grade data and skewed data. Count data were expressed as the number of cases or percentage (%), and were compared by Chi-square test or Fisher's exact method. *P* < 0.05 was considered statistically significant. SPSS 20.0 software was used for analysis.

## Results

**Fig. 1:**
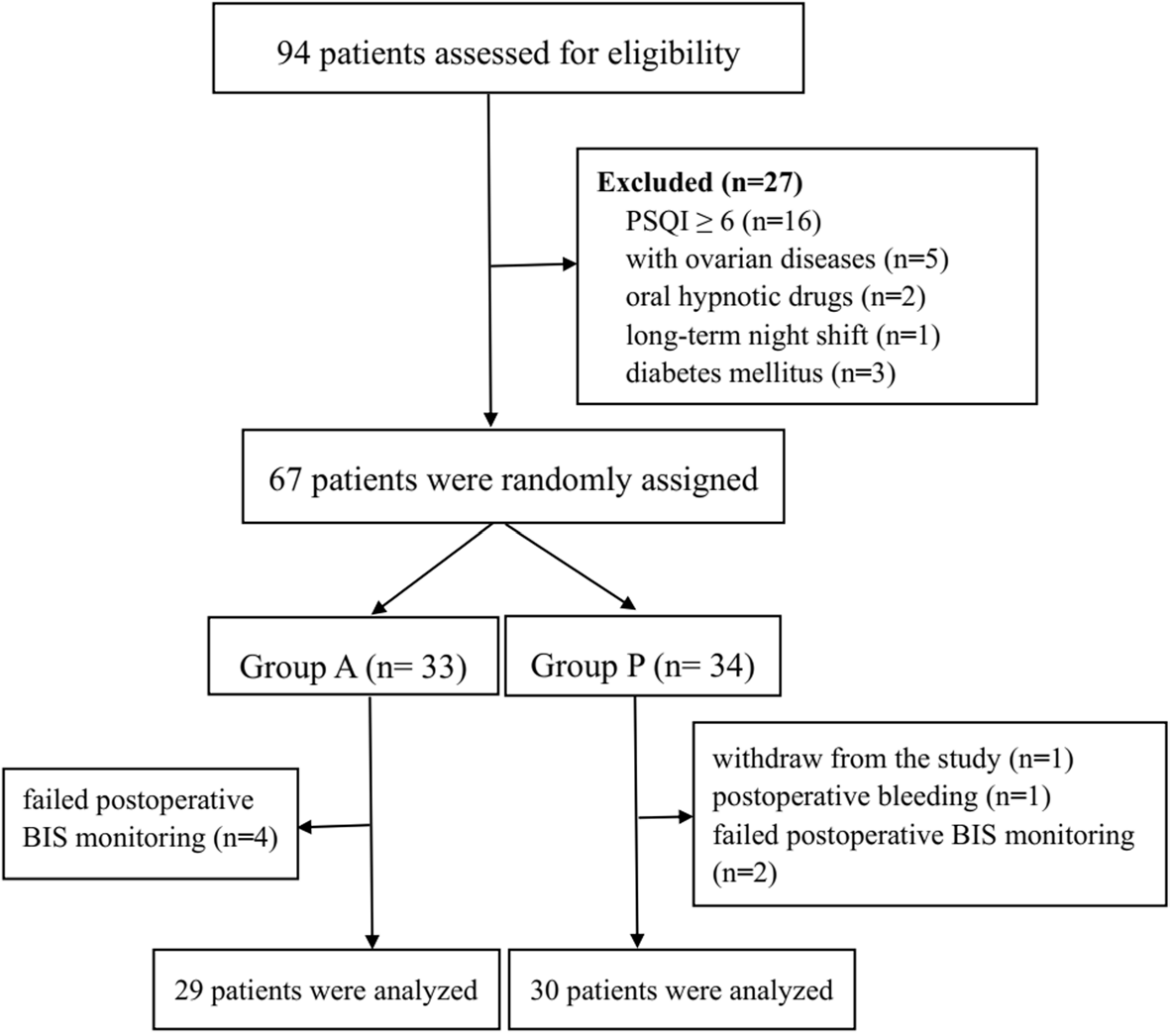
Flow diagram showing the patients that were included and excluded in this study

Based on the inclusion criteria, 94 patients were selected, and all surgeries were performed by the same group of surgeons (Fig. 1). Finally, there were 29 patients in group A and 30 patients in group P for statistical analysis. No statistically significant differences were noted in the age, body mass index, ASA classification, operation time, anesthesia time, PACU recovery time, and distribution of anesthetic drugs used between the groups (*P* > 0.05), (Table 1).

**Table 1:** Comparison of the general conditions of the two groups of patients

Items	Evaluation index	Group A	Group P	*P* value
Basic information	Age (years, mean ± SD)	42.0 ± 6.6	41.0 ± 5.5	0.418
	BMI (kg/m^2^, mean ± SD)	23.3 ± 2.1	23.8 ± 2.4	0.312
	Years of education (years, mean ± SD)	7.1 ± 3.1	7.3 ± 3.4	0.498
	ASA I /II (cases)	7/22	6/24	0.761
	Hemoglobin (g/dl, mean ± SD)	11.3 ± 1.2	11.0 ± 1.1	0.852
	Hypertension [cases (%)]	5 (17%)	7 (23%)	0.748
	postmenopause/paramenia/ normal menstruation(cases)	8/9/12	6/10/14	0.788
	Operation time (min)	74.6 ± 24.3	69.6 ± 20.1	0.521
	NPO (h)	10.1 ± 1.9	8.9 ± 1.7	0.213
Situation of operation (mean ± SD)	Anesthesia time (min)	100.2 ± 30.6	101.7 ± 31.2	0.368
	PACU time (min)	38.3 ± 8.0	37.4 ± 4.8	0.830
	Blood loss (ml)	132.5 ± 32.9	121 ± 30.2	0.440
	Urine volume (ml)	200.4 ± 51.0	234.5 ± 56.5	0.768
Anesthetics (mean ± SD)	Sufentanil (μg)	27.7 ± 3.4	28.2 ± 3.0	0.555
	Remifentanil (μg)	956.5 ± 375.1	914.5 ± 343.3	0.646
	Rocuronium (mg)	47.4 ± 11.5	49.7 ± 14.7	0.503
	Propofol (mg)	354.5 ± 92.0	362.6 ± 89.7	0.728
Postoperative management	Antiemetic treatment [cases (%)]	5 (17%)	4 (13%)	0.730
	Remedial analgesia [cases (%)]	5 (17%)	5 (16%)	0.953

There were no significant differences between the two groups of patients in the preoperative total scores of AIS and the scores of each part (*P* > 0.05). Compared with the one day before the operation, the total AIS scores, overall quality of sleep, total sleep duration, and final awakening earlier than desired scores of the two groups of patients were significantly increased on the first day after the operation (*P* < 0.01). Compared with the one day before the operation, the sleep induction and physical and mental functioning during the day scores on the first day after surgery in group P increased significantly (*P* < 0.05), but the sleep induction and physical and mental functioning during the day scores on the first day after surgery in group A did not change significantly (*P* > 0.05). Compared with group A on the first day after surgery, the total AIS scores, overall quality of sleep, sleep induction, total sleep duration, final awakening earlier than desired, and physical and mental functioning during the day scores of group P increased significantly (*P* < 0.05), Table 2. The incidence of postoperative sleep disturbances in group P was significantly higher than that in group A (70.0% *vs.* 37.9%, *P* < 0.05).

**Table 2:** The scores of each item on the AIS scale of the two groups of patients

	Group A (*n* = 29)		Group P (*n* = 30)	
	Before surgery	After surgery	Before surgery	After surgery
**AIS**	3 (2,3)	5 (4,8) ^a^	3 (1,4)	9 (8,11) ^bc^
**Overall quality of sleep**	0 (0,1)	1 (1,1) ^a^	1 (0,1)	1 (1,2) ^bc^
**Sleep induction**	1 (0,1)	1 (0,1)	1 (0,1)	1 (1,2) ^bc^
**Total sleep duration**	0 (0,1)	1 (0,2) ^a^	0 (0,0)	3 (2,3) ^bc^
**Final awakening earlier than desired**	0 (0,0)	1 (1,2) ^a^	0 (0,0)	3 (2,3) ^bc^
**Awakenings during the night**	1 (0,1)	1 (1,1)	1 (0,1)	1 (1,1)
**Sense of well-being** **during the day**	0 (0,0)	0 (0,0)	0 (0,0)	0 (0,0)
**Functioning during the day**	0 (0,0)	0 (0,0)	0 (0,1)	1 (0,1) ^bc^
**Sleepiness during the day**	0 (0,0)	0 (0,0)	0 (0,0)	0 (0,0)

^a^*P* < 0.05, vs group A 1 day after operation

^b^*P* < 0.05, vs group P 1 day before operation

^c^*P* < 0.05, vs group A 1 day after operation

**Fig. 2:**
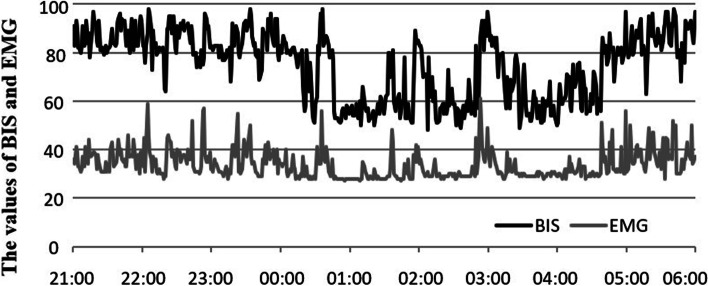
Typical trends of BIS and electromyogram changes in group A patients on the first night under BIS monitoring

**Fig. 3:**
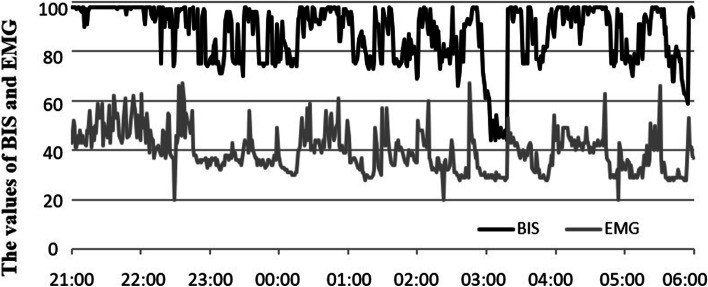
Typical trends of BIS and electromyogram changes in group P patients on the first night under BIS monitoring

Compared with group A, the total sleep duration under BIS monitoring in group P was significantly shorter (*P* < 0.01). The sleep efficiency (*P* < 0.01), and the overall quality of sleep (BIS-AUC) (*P* < 0.01) was significantly reduced, Table 3. The typical trends of BIS and frontal muscle electromyogram changes of the two groups of patients under BIS monitoring on the first night after surgery are shown in Figs. 2 and 3.

**Table 3:** Comparison of sleep status under BIS monitoring on the first night after operation between the two groups

	**Sleep duration (min)**	**Sleep efficacy (%)**	**BIS-AUC (%)**
**Group A (*n* = 29)**	245.5 ± 77.2	46.0 ± 14.2	77.7 ± 4.7
**Group P (*n* = 30)**	168.0 ± 75.4 ^a^	31.4 ± 14.1 ^a^	84.0 ± 4.2 ^a^

^a^*P* < 0.01, vs group A 1 day after operation

There were no significant differences in the levels of MT, cortisol, blood glucose, and pain between the two groups before surgery (*P* > 0.05); compared with one day before surgery, the postoperative cortisol, blood glucose, and pain levels of the two groups increased significantly, while the MT level was significantly reduced (*P* < 0.05); compared with group A, postoperative cortisol was significantly increased and MT secretion was significantly reduced in group P (*P* < 0.05), Table 4.

**Table 4:** Comparison of stress response indicators between the two groups of patients

		**Melatonin (pg/ml)**	**Cortisol (ng/ml)**	**Blood glucose (mmol/L)**	**Pain**
Group A (*n* = 29)	**1d before surgery**	252.1 ± 37.9	184.7 ± 46.8	3.84 ± 0.74	1 (0,2)
	**1d after surgery**	210.0 ± 42.0^a^	248.0 ± 26.6^a^	5.92 ± 1.40^a^	2 (1,2) ^a^
Group P (***n* = 30**)	**1d before surgery**	262.3 ± 52.0	186.4 ± 48.6	4.20 ± 0.72	1 (0,2)
	**1d after surgery**	154.5 ± 43.6^ab^	291.2 ± 46.5^ab^	6.48 ± 1.20^a^	2 (2,3) ^a^

^a^*P* < 0.05, vs preoperative

^b^*P* < 0.05, vs group A 1 day after operation

## Discussion

POSD is one of the most common postoperative brain dysfunctions. The incidence of POSD can be as high as 42% [8], especially on the first night after surgery, reducing the patients’ total sleep duration by 80% [2]. Studies have shown that the worse the overall quality of sleep of patients on the first postoperative night, the longer the postoperative hospital stay. The quality of sleep on the first postoperative night is an important factor affecting the patient's postoperative recovery [9]. The main factors affecting the postoperative sleep function include: advanced age, major surgery, heavy bleeding, postoperative pain, anesthetic, environmental, psychological, and mental factors [10]. Since, elderly patients are prone to sleep disorders, to minimize the influence of the age factor on the result, this study included only young and middle-aged patients. Further this study included only laparoscopic myomectomy surgery, as it is a minimally invasive, shorter operation time, less bleeding, less postoperative pain with fewer postoperative complications, and less psychological pressure on patients in an aim to exclude the interference of the surgical factor and postoperative pain. Except for the difference in the start time of surgical anesthesia, the general information, surgical duration, and anesthesia medications, incidence of postoperative nausea, and analgesic remediation of the two groups of patients were all comparable.

The results of this study showed that the overall quality of sleep, total sleep duration, and early awakening of the two groups of patients on the first night after surgery were significantly lower than those before surgery. These results concur studies showing prolonged sleep induction, shortened sleep duration, increased number of awakenings, and fragmented sleep after surgery [11, 12]. Secretion of MT influences circadian rhythm and is closely related to the sleep rhythm. Under normal sleep function, MT secretion is strengthened at night, reaching a peak at 2–3 am; when it is turned to a bright environment, the secretion is stopped. A decrease in MT level directly affects the quality of sleep, resulting in prolonged sleep induction, significantly increased awakenings, and decreased sleep quality [13]. Anesthesia for surgery can seriously disrupt postoperative sleep by delaying the secretion of MT at night. Secretion of MT on the first night after surgery is significantly lower than that before surgery [14], and with no secretion detected even at 10 pm in patients undergoing hysterectomy [15]. MT secretion was significantly lower than that before surgery in the two groups of patients, especially in the afternoon surgery group. The change in sleep function on the first night after surgery shows that even general anesthesia for minimally invasive surgery can interfere with the patient's sleep function immediately after surgery.

This study focused on comparing the effects of anesthesia for morning and afternoon surgeries on the sleep function of patients on the first night after surgery. The results showed that the sleep function on the first night after surgery in the afternoon surgery group was significantly worse than that in the morning surgery group. The number of night awakenings in the afternoon surgery group was significantly greater than that in the morning surgery group. The incidence of postoperative sleep disturbance in the afternoon surgery group was significantly higher than that in the morning surgery group (70.0% *vs.* 37.9%). These results indicate that compared with the morning surgery group, the afternoon surgery group is not conducive to the restoration of sleep function during the early postoperative period. Studies have confirmed that the circadian rhythm has a significant impact on the intensity of intravenous anesthetics, the amount of anesthetics required in the morning and during the active period is relatively large, which may be related to the faster liver and kidney metabolism during this period [16]. In this study, the two groups of patients had a short anesthesia time and the consumption of anesthetic drugs was similar, and thus the metabolism of anesthetic drugs in the morning surgery group should be more complete than that in the afternoon group. Patients in the morning surgery group had a longer time to adjust and recover the body rhythm, and the brain function of patients in the morning surgery group might recover faster. Opioids have been shown to significantly interfere with the sleep structure of patients [9, 17]. Sufentanil has a longer duration, and it is more likely to accumulate in patients in the afternoon surgery group. Therefore, it may seriously interfere with sleep at night for patients who undergo surgery in the afternoon.

Anesthesia for surgery is used as a stress response source, and the magnitude of the stress response will also interfere with the secretion level and cycle of MT [18]. In the present study, the cortisol level in the morning on the first postoperative day was higher in the afternoon surgery group than that in the morning surgery group, and the level of MT in the blood in the afternoon surgery group on the first day after surgery was lower than that in the morning surgery group. This result also supports that the stress response in the afternoon surgery group on the first night after surgery is stronger than that in the morning surgery group, and the suppression of MT secretion at night is more obvious in the afternoon surgery group, which affects the recovery of early postoperative sleep function. Blood glucose and pain are also important indicators of stress levels. In this study, there was no significant difference in the postoperative blood glucose level between the two groups of patients, which may be related to the fact that all patients in this study were middle-aged and young people who have similar recovery and adjustment ability, and the surgical trauma was relatively mild. There was no significant difference in the postoperative night pain between the two groups of patients, which was related to adequate postoperative analgesia, and it also ruled out the effect of pain on postoperative sleep. Animal experiments have shown that the MT secreted by the pineal gland and its rhythm of activity have opposite phase changes when ketamine anesthesia is given at different time points (rest period and exercise period) [19]. Despite only the morning and afternoon operations are compared in this study, but the results still indicate that the MT secretion is inhibited more significantly and the night sleep function is worse on the first night after surgery in the afternoon surgery group. This study used a BIS monitoring device to objectively evaluate the sleep function on the first night after surgery, the results also confirmed that the total sleep duration, sleep efficiency, and overall quality of sleep of patients in the afternoon surgery group was lower than those in the morning surgery group.

Limitations of this study: (1) This study only compared the morning and afternoon surgery groups and failed to compare more detailed groups including the night surgery group. (2) This study did not use the "gold standard", polysomnography, for evaluating sleep function. This monitoring requires a special venue and is not conducive to the monitoring of the sleep function of patients after surgery. Therefore, the sleep scale and BIS monitoring were combined to evaluate the sleep function. Studies have shown that BIS monitoring has a good correlation with the patients' sleep [20]. (3) This study did not dynamically evaluate the changes in the secretion phase and secretion level of the patient's MT, mainly considering that multiple blood sample collection at night also interferes with the patient's sleep [21]. (4) Menstruation affects sleep [22], but many of our patients have menopause or menstrual disorders, so there is no way to accurately evaluate the specific impact of menstrual cycle on sleep. Nevertheless, we still distinguished the number of menopause, menstrual disorders and normal menstruation in the two groups, and there was no significant difference between them.

## Conclusion

In conclusion, we found that the sleep function (including sleep time, sleep efficiency, sleep quality, etc.) of patients undergoing general anesthesia decreased significantly on the first night after surgery. Especially in the afternoon, patients with surgery had stronger stress, more obvious melatonin inhibition and worse sleep function than those in the morning. Therefore, we suggest that the potential impact of surgery time on sleep should be considered for some high-risk individuals who may develop postoperative sleep disturbances. In future studies, other surgical types, broader populations, and longer periods of sleep should be researched.

## Data Availability

The datasets used and analysed during the current study are available from the corresponding author on reasonable request.

## Abbreviations

POSD: Postoperative sleep disturbances
PSQI: Pittsburgh sleep quality index
AIS: Athens Insomnia Scale
BIS: Bispectral index
MT: Melatonin
ASA: American society of anaesthesiologists
PACU: Postanesthesia care unit
